# Supplementary material for: Geographic name resolution service: A tool for the standardization and indexing of world political division names, with applications to species distribution modeling
Source: PLoS One. 2022 Nov 14;17(11):e0268162. doi: 10.1371/journal.pone.0268162 (PMC9662723; doi:10.1371/journal.pone.0268162)
Supplement: S1 Appendix — (PDF) [file pone.0268162.s001.pdf]

S1 Appendix: GNRS Performance features

Several features of the GNRS enable it to process large requests from multiple users very rapidly . One approach involves careful use of Postgres indexes, including heavy indexing of read-only tables and the extensive use of partial indexes.

Caching of previous results also accelerates performance. The benefits of caching increase over time as more data are processed. After processing of a given batch is complete, name resolution results for any political divisions not previously processed are saved to table "cache". Before processing a new batch of political divisions, the GNRS checks if any submitted political divisions are already in the cache. If so, the cached results are loaded to the user data table and omitted from further processing. Caching dramatically reduces processing time for batches consisting largely of political divisions with resolution results already in the cache. The cache is cleared whenever the database is rebuilt or changes are made to GNRS code that can affect resolution results.

Parallel processing increases processing speed which scale approximately linearly with the number of CPUs. When the GNRS runs in parallel mode (the default for the GNRS API), incoming requests are managed by a parallelization controller written in perl. The controller breaks large requests into multiple smaller batches, which are then submitted as gnrs_batch commands using makeflow (Albrecht et al. 2011). Makeflow distributes batches among available cores and monitors processing until all batches have been completed, after which the batches are reassembled into a single output file.

References

Albrecht, Michael, Patrick Donnelly, Peter Bui, and Douglas Thain. 2011. "Makeflow: A Portable Abstraction for Cluster, Cloud, and Grid Computing." *Technical Report TR-2011--02*. http://www.cse.nd.edu/Reports/2011/TR-2011-02.pdf.
