# Supplementary material for: Geographic name resolution service: A tool for the standardization and indexing of world political division names, with applications to species distribution modeling
Source: PLoS One. 2022 Nov 14;17(11):e0268162. doi: 10.1371/journal.pone.0268162 (PMC9662723; doi:10.1371/journal.pone.0268162)
Supplement: S2 Appendix — (PDF) [file pone.0268162.s002.pdf]

S2 Appendix: Building the GNRS reference database

Merging the reference data

In linking political divisions from GADM to GeoNames, we first matched by codes (ISO, FIPS, and HASC) given their greater standardization relative to names and, therefore lower risk of spurious matches. As no code system was shared between GADM and GeoNames below the country level, we used NaturalEarth as a crosswalk table, joining names from NaturalEarth to Geonames by geoname_id (also included in the NaturalEarth database) and from NaturalEarth to GADM by HASC codes. For political divisions that could not be matched by code, we performed case-insensitive exact matching to standard and alternative names, with accented characters converted to plain ascii equivalents. For the remaining unmatched names, we searched for and removed class identifiers and related parts of speech such as “State”, “State of”, “Province de”, “Municipio de”, “Oblast”, etc., after which we re-matched the remaining base names. The political division hierarchy constrained matching: countries were matched first, followed by first-level political divisions within countries, then second-level political divisions within first-level divisions.

To avoid introducing errors, we minimized the use of wildcards and fuzzy matching while building the reference database. However, during the initial build of the GNRS database we used wildcards to search manually for near matches among the remaining unmatched names. Unmatched names discovered and verified during manual searches were then hard-coded into the database pipeline so they can be included automatically in future rebuilds.

Not all political divisions could be aligned between Geonames and GADM. Unmatched political divisions are identifiable in the GNRS database by having either a Geonames or a GADM identifier but not both. At the country level, we matched all countries except Kosovo (ISO Xk), which was present in Geonames but not in GADM. At the state level, all 3,616 states in GADM matched a Geonames state but 765 states in Geonames did not match a corresponding state in GADM. At the county level, 45,660 political divisions matched between the two databases, 236 were unique to GADM, and 12,611 were unique to Geonames.

Much of the match asymmetry between GADM and GeoNames is due to the greater number of political division levels in GeoNames. For example, for Sri Lanka, only two levels of the political division below the country level (district and division) are represented in GADM. In contrast, Geonames also includes Sri Lankan provinces, which sit below the country level but above the “division” level in that country’s political division hierarchy. Although provinces were given legal status in Sri Lanka in 1987 in the Indo-Sri Lanka Accord (Marasinghe 1988), they are not represented in GADM. Despite these non-matches, the primary goal of the GNRS—to resolve submitted political divisions to GADM spatial objects—has been met. The remaining 236 unlinked level 2 GADM political divisions will be resolved in future releases of the GNRS database.

References

Marasinghe, M. L. 1988. “Ethnic Politics and Constitutional Reform: The Indo-Sri Lankan Accord.” *The International and Comparative Law Quarterly* 37 (3). Cambridge University Press: 551–87.
