# Supplementary material for: Geographic name resolution service: A tool for the standardization and indexing of world political division names, with applications to species distribution modeling
Source: PLoS One. 2022 Nov 14;17(11):e0268162. doi: 10.1371/journal.pone.0268162 (PMC9662723; doi:10.1371/journal.pone.0268162)
Supplement: S3 Appendix — (PDF) [file pone.0268162.s003.pdf]

S3 Appendix: Administrative division hierarchy statistics for GADM and GBIF

Administrative divisions per administrative division level in GADM

To obtain counts of the numbers of administrative divisions at each level of the administrative division hierarchy in the Global Administrative Areas (GADM) Database, we imported a download of GADM version 3.6 to PostgreSQL database ”gadm”, according to the steps described in (Boyle 2022a). Counts were obtained using the following SQL commands.

Table 1. SQL to extract counts of administrative divisions at each level of the administrative division hierarchy in GADM. Postgres responses are included in this example to show the values returned. For this reason, be careful to paste only SQL code into the Postgres console when attempting to execute this code.

```
\c gadm

-- country (level 1)
SELECT COUNT(DISTINCT gid_0) FROM gadm;
 count
-------
   256
(1 row)

 -- admin_1 (level 2)
SELECT COUNT(DISTINCT gid_1) FROM gadm;
 count
-------
  3610
(1 row)

-- admin_2 (level 3)
SELECT COUNT(DISTINCT gid_2) FROM gadm;
 count
-------
 45962
(1 row)

-- admin_3 (level 4)
SELECT COUNT(DISTINCT gid_3) FROM gadm;
 count
--------
 147427
(1 row)

-- admin_4 (level 5)
SELECT COUNT(DISTINCT gid_4) FROM gadm;
 count
```

```
--------
 138053
(1 row)

-- admin_5 (level 6)
SELECT COUNT(DISTINCT gid_5) FROM gadm;
 count
-------
 51427
(1 row)

-- Total poldivs level 3 and lower
SELECT COUNT(DISTINCT gid_0) + COUNT(DISTINCT gid_1) + COUNT(DISTINCT gid_2)
AS tot_poldivs_levels_1to3
FROM gadm
;
 tot_poldivs_levels_1to3
-------------------------
                   49828
(1 row)

-- Total poldivs level 4 and higher
SELECT COUNT(DISTINCT gid_3) + COUNT(DISTINCT gid_4) + COUNT(DISTINCT gid_5)
AS tot_poldivs_levels_4to6
FROM gadm
;
 tot_poldivs_levels_4to6
-------------------------
                  336907
(1 row)
```

Counts of GBIF occurrences by political division level

To obtain counts of GBIF species occurrences by political divisions level, we first downloaded and exported all plant occurrences in GBIF (GBIF.org 2020) and imported the download to Postgres, according to the instructions in (Boyle 2022b). Next, to build summary tables, we executed the following commands in the GBIF database:

Table 2. SQL to obtain counts of occurrences by declared political division level in a download of all plant occurrences in GBIF. As Postgres responses are not included, this code can be executed directly by pasting into the Postgre console.

```
\c GBIF

DROP TABLE IF EXISTS gbif_poldiv_levels_raw;
CREATE TABLE gbif_poldiv_levels_raw AS
SELECT "gbifID",
```

```
"countryCode", "stateProvince", "county", "municipality",
CASE
WHEN "countryCode" IS NULL THEN 0
ELSE 1
END AS has_country,
CASE
WHEN "stateProvince" IS NULL THEN 0
ELSE 1
END AS has_state,
CASE
WHEN county IS NULL THEN 0
ELSE 1
END AS has_county,
CASE
WHEN municipality IS NULL THEN 0
ELSE 1
END AS has_municipality
FROM occurrence
;

-- Index admin level summary field
CREATE INDEX gbif_poldiv_levels_raw_has_country_idx ON
gbif_poldiv_levels_raw(has_country);
CREATE INDEX gbif_poldiv_levels_raw_has_state_idx ON
gbif_poldiv_levels_raw(has_state);
CREATE INDEX gbif_poldiv_levels_raw_has_county_idx ON
gbif_poldiv_levels_raw(has_county);
CREATE INDEX gbif_poldiv_levels_raw_has_municipality_idx ON
gbif_poldiv_levels_raw(has_municipality);


ALTER TABLE gbif_poldiv_levels_raw
ADD COLUMN poldiv_level text,
ADD COLUMN tot_poldiv_levels integer
;

UPDATE gbif_poldiv_levels_raw
SET poldiv_level=
CASE
WHEN has_country=1 AND has_state=1 AND has_county=1 AND has_municipality=1
THEN 'municipality'
WHEN has_country=1 AND has_state=1 AND has_county=0 AND has_municipality=1
THEN 'municipality (no county)'
WHEN has_country=1 AND has_state=1 AND has_county=1 AND has_municipality=0
THEN 'county (no municipality)'
WHEN has_country=1 AND has_state=1 AND has_county=0 AND has_municipality=0
THEN 'state'
WHEN has_country=1 AND has_state=0 AND has_county=0 AND has_municipality=0
THEN 'country'
WHEN has_country=0 AND has_state=0 AND has_county=0 AND has_municipality=0
THEN 'none'
ELSE 'other'
END
;

UPDATE gbif_poldiv_levels_raw
```

```
SET tot_poldiv_levels=
CASE
WHEN poldiv_level='municipality' THEN 4
WHEN poldiv_level='county (no municipality)' OR poldiv_level='municipality
(no county)' THEN 3
WHEN poldiv_level='state' THEN 2
WHEN poldiv_level='country' THEN 1
WHEN poldiv_level='none' THEN 0
ELSE NULL
END
;

-- Index primary keys in case need to join back to original table
--CREATE INDEX occurrence_gbif_id_idx ON occurrence("gbifID");
--CREATE INDEX gbif_poldiv_levels_raw_gbif_id_idx ON
gbif_poldiv_levels_raw("gbifID");

--
-- Political division levels
--

DROP TABLE IF EXISTS gbif_poldiv_levels;
CREATE TABLE gbif_poldiv_levels AS
SELECT poldiv_level, COUNT(*) AS obs
FROM gbif_poldiv_levels_raw
GROUP BY poldiv_level
;

ALTER TABLE gbif_poldiv_levels
ADD COLUMN perc_obs decimal(5,2)
;

UPDATE gbif_poldiv_levels
SET perc_obs = (
obs/(SELECT SUM(obs) FROM gbif_poldiv_levels) * 100
)::decimal(5,2)
;

--
-- Political division level counts
--

DROP TABLE IF EXISTS gbif_tot_poldiv_levels;
CREATE TABLE gbif_tot_poldiv_levels AS
SELECT tot_poldiv_levels, COUNT(*) AS obs
FROM gbif_poldiv_levels_raw
GROUP BY tot_poldiv_levels
;

ALTER TABLE gbif_tot_poldiv_levels
ADD COLUMN perc_obs decimal(5,2)
;

UPDATE gbif_tot_poldiv_levels
SET perc_obs = (
obs/(SELECT SUM(obs) FROM gbif_tot_poldiv_levels) * 100
```

```
)::decimal(5,2)
;
```

References

Boyle, Brad. 2022a. *Gadm: Build GADM (Global Administrative Areas) PostgreSQL Database*. Github. Accessed August 23. https://github.com/ojalaquellueva/gadm.

———. 2022b. *import_GBIF: Example Import of GBIF Download to PostgreSQL*. Github. Accessed August 23. https://github.com/ojalaquellueva/import_GBIF.

GBIF.org. 2020. “GBIF Occurrence Download.” The Global Biodiversity Information Facility. doi:10.15468/DL.87ZYEZ.
