# Supplementary material for: Geographic name resolution service: A tool for the standardization and indexing of world political division names, with applications to species distribution modeling
Source: PLoS One. 2022 Nov 14;17(11):e0268162. doi: 10.1371/journal.pone.0268162 (PMC9662723; doi:10.1371/journal.pone.0268162)
Supplement: S4 Appendix — (PDF) [file pone.0268162.s004.pdf]

## S4 Appendix: States-as-countries and countries-as-states

Two classes of political divisions not resolved by the default GNRS workflow required custom solutions. These are (1) territories and other sub-national geopolitical units treated as countries ("states-as-countries") by the reference databases, and (2) countries belonging to multinational unions, with the countries treated as first-level divisions (e.g., states) and the unions treated as countries ("countries-as-states").

An example of states-as-countries is Puerto Rico, an unincorporated territory of the United States. In GADM, as in most geopolitical databases, Puerto Rico is treated as a top-level political division with ISO code PR. Biodiversity observations in which Puerto Rico appears as a US state (e.g., "USA, Puerto Rico, Mayaguez") are resolved to the country only ("United States") using the default GNRS name resolution workflow. To remedy this issue, we added a recursive link from all "states-as-countries" within the GNRS country table to the alternative containing country. For political divisions which do not resolve at the state level, the GNRS checks if the submitted state appears in the country table linked recursively to another country. If so, the original submitted country is removed and the state and 2nd-level political division, if any, are resolved as country and state. For the preceding example, "USA, Puerto Rico, Mayaguez" is resolved to "Puerto Rico, Mayaguez". Special handling of states-as-countries is essential for resolving islands, island groups, semi-autonomous regions and protectorates which are treated as top-level administrative divisions in geospatial reference databases but sometimes listed under the associated country in biodiversity database (e.g., Aland Islands / Finland, British Virgin Islands / UK, US Virgin Islands / USA, Bouvet Island / Denmark, Clipperton Island / France). The GNRS does not take a stand concerning the official status and jurisdiction of such political divisions; rather, it searches alternative classifications to match all possible usages to the particular version used by the reference database.

An example of countries-as-states is Scotland. Along with other member countries of the United Kingdom, Scotland is treated as a 1st-level political division the ("state/province") in the GADM database. Its parent entity, the United Kingdom, is treated as a country, with ISO code UK. In the GNRS database, such countries are stored in the table state_province and flagged as a country-as-state (country_as_state=1); the parent entity is stored in the country table. To detect such cases, unresolved countries are checked against all political entities in the state_province table flagged as "country-as-state. Any matches found are resolved at the state/province level and flagged "country-as-state" in field match_method_state_province; the containing political entity (in the case of Scotland, "United Kingdom"), is resolved as a country and flagged "inferred from country-as-state" in field match_method_country. Thus, "Scotland,Perthshire,Aberfeldy" resolves to "United Kingdom,Scotland,Perthshire and Kinross"; the original 2nd-level division (Aberfeldy) is not resolved. Other countries resolved by this method are England, Northern Ireland and Wales.
