# Supplementary material for: Geographic name resolution service: A tool for the standardization and indexing of world political division names, with applications to species distribution modeling
Source: PLoS One. 2022 Nov 14;17(11):e0268162. doi: 10.1371/journal.pone.0268162 (PMC9662723; doi:10.1371/journal.pone.0268162)
Supplement: S5 Appendix — (PDF) [file pone.0268162.s005.pdf]

S5 Appendix: Complete input-output for "Example: Sample workflow with the GNRS R package"

Example 1: A few political divisions

GNRS_super_simple() is the quickest method of standardizing a small number of political division names. This function does not require that the user supply a dataframe and instead takes character vectors as input. Complete output from this example is provided in library GNRS.

Table 1. Examples of resolving a individual political divisions.

```
library("GNRS")

GNRS_super_simple("USA")

##   poldiv_full country_verbatim state_province_verbatim
## 1       USA@@              USA
##   state_province_verbatim_alt county_parish_verbatim county_parish_verbatim_alt
## 1
##         country state_province county_parish country_id state_province_id
## 1 United States                               6252001
##   county_parish_id country_iso state_province_iso county_parish_iso geonameid
## 1                          US                                     6252001
##   gid_0 gid_1 gid_2 match_method_country match_method_state_province
## 1   USA                 iso_alpha3 code
##   match_method_county_parish match_score_country match_score_state_province
## 1                                       1.00
##   match_score_county_parish overall_score poldiv_submitted poldiv_matched
## 1                                 1.00          country        country
##   match_status user_id
## 1   full match       1

GNRS_super_simple(country = c("USA", "Canada"))

##   poldiv_full country_verbatim state_province_verbatim
## 1       USA@@              USA
## 2    Canada@@           Canada
##   state_province_verbatim_alt county_parish_verbatim county_parish_verbatim_alt
## 1
## 2
##         country state_province county_parish country_id state_province_id
## 1 United States                               6252001
## 2        Canada                               6251999
##   county_parish_id country_iso state_province_iso county_parish_iso geonameid
## 1                          US                                     6252001
## 2                          CA                                     6251999
##   gid_0 gid_1 gid_2 match_method_country match_method_state_province
## 1   USA                 iso_alpha3 code
## 2   CAN             exact standard name
##   match_method_county_parish match_score_country match_score_state_province
```

```
## 1                                      1.00
## 2                                      1.00
##   match_score_county_parish overall_score poldiv_submitted poldiv_matched
## 1                         1.00          country        country
## 2                         1.00          country        country
##   match_status user_id
## 1   full match       1
## 2   full match       2
```

```
GNRS_super_simple(country = "USA",
                  state_province = "AZ",
                  county_parish = "Pima County")
```

```
##         poldiv_full country_verbatim state_province_verbatim
## 1 USA@AZ@Pima County              USA                      AZ
##   state_province_verbatim_alt county_parish_verbatim county_parish_verbatim_alt
## 1                                        Pima County                       Pima
##        country state_province county_parish country_id state_province_id
## 1 United States        Arizona          Pima    6252001           5551752
##   county_parish_id country_iso state_province_iso county_parish_iso geonameid
## 1          5308878          US                 AZ               019   5308878
##   gid_0   gid_1      gid_2 match_method_country match_method_state_province
## 1   USA USA.3_1 USA.3.11_1      iso_alpha3 code                    iso code
##   match_method_county_parish match_score_country match_score_state_province
## 1                exact name                1.00                       1.00
##   match_score_county_parish overall_score poldiv_submitted poldiv_matched
## 1                      1.00          1.00    county_parish  county_parish
##   match_status user_id
## 1   full match       1
```

Example 2: Many political divisions

In most cases, users will have existing data sets containing political division names that they wish to standardize. In this case, the user only has to generate an appropriately formatted dataframe from their dataset. This can be done manually, or the function GNRS_template() can be used to generate an empty dataframe that can then be populated. Here, we demonstrate this using the data packaged with the GNRS R package (accessed through the data() function).

Table 2. Example of resolving files of many political divisions.

```
data("gnrs_testfile")

gnrs_dataframe <- GNRS_template(nrow = nrow(gnrs_testfile))

gnrs_dataframe$country <- gnrs_testfile$country

gnrs_dataframe$state_province <- gnrs_testfile$state_province

gnrs_dataframe$county_parish <- gnrs_testfile$county_parish

clean_dataframe <- GNRS(political_division_dataframe = gnrs_dataframe)
```

```
head(clean_dataframe)
```

```
##                             poldiv_full country_verbatim
## 1       Russia@Lipetsk@Dobrovskiy rayon           Russia
## 2 Mexico@Sonora, Estado de@Hua^sA(C)pac           Mexico
## 3                     Guatemala@Izabal@        Guatemala
## 4               USA@Arizona@Pima County              USA
## 5                    U.S.A@Arizona@Pima            U.S.A
## 6                          USA@Ilinois@              USA
##   state_province_verbatim state_province_verbatim_alt county_parish_verbatim
## 1                 Lipetsk                                   Dobrovskiy rayon
## 2       Sonora, Estado de                                       Hua^sA(C)pac
## 3                  Izabal
## 4                 Arizona                                        Pima County
## 5                 Arizona                                               Pima
## 6                 Ilinois
##   county_parish_verbatim_alt       country     state_province    county_parish
## 1                 Dobrovskiy        Russia Lipetskaya Oblast' Dobrovskiy Rayon
## 2                                   Mexico             Sonora
## 3                                Guatemala             Izabal
## 4                       Pima United States            Arizona             Pima
## 5                            United States            Arizona             Pima
## 6                            United States           Illinois
##   country_id state_province_id county_parish_id country_iso state_province_iso
## 1    2017370            535120           565805          RU                 43
## 2    3996063           3982846                           MX                 26
## 3    3595528           3595259                           GT                 09
## 4    6252001           5551752          5308878          US                 AZ
## 5    6252001           5551752          5308878          US                 AZ
## 6    6252001           4896861                           US                 IL
##   county_parish_iso geonameid gid_0    gid_1      gid_2 match_method_country
## 1            565805    565805   RUS RUS.39_1 RUS.39.5_1  exact standard name
## 2                     3982846   MEX MEX.26_1             exact standard name
## 3                     3595259   GTM  GTM.9_1             exact standard name
## 4               019   5308878   USA  USA.3_1 USA.3.11_1      iso_alpha3 code
## 5               019   5308878   USA  USA.3_1 USA.3.11_1 fuzzy alternate name
## 6                     4896861   USA USA.14_1                 iso_alpha3 code
##   match_method_state_province match_method_county_parish match_score_country
## 1        exact alternate name                exact name                 1.00
## 2           wildcard alt name                                           1.00
## 3      exact ascii short name                                           1.00
## 4                  exact name                exact name                 1.00
## 5                  exact name     exact ascii short name                 1.00
## 6           wildcard alt name                                           1.00
##   match_score_state_province match_score_county_parish overall_score
## 1                       1.00                      1.00          1.00
## 2                       1.00                      0.00          0.67
## 3                       1.00                                    1.00
## 4                       1.00                      1.00          1.00
## 5                       1.00                      1.00          1.00
## 6                       1.00                                    1.00
##   poldiv_submitted poldiv_matched  match_status user_id
## 1    county_parish  county_parish    full match       1
## 2    county_parish state_province partial match       2
## 3   state_province state_province    full match       3
## 4    county_parish  county_parish    full match       4
## 5    county_parish  county_parish    full match       5
## 6   state_province state_province    full match       6
```

In both use cases, the function GNRS_metadata() is usually the last step and is used to extract information that is needed for publication (e.g. version number, citation information).

Table 3. Examples of GNRS metadata requests

```
metadata <- GNRS_metadata()

metadata$sources

##   source_id   source_name                          source_name_full
## 1         2          GADM Database of Global Administrative Areas
## 2         3 Natural Earth                          Natural Earth Data
## 3         1      geonames          GeoNames geographical database
##                         source_url
## 1                 https://gadm.org/
## 2 https://www.naturalearthdata.com/
## 3              https://geonames.org
##
description
## 1 GADM, the Database of Global Administrative Areas, is a high-resolution
database of country administrative areas, with a goal of "all countries, at all
levels, at any time period.
## 2
Lookup tables of admin 1 (state/province) names and codes. From Natural Earth Data,
## 3                                The GeoNames geographical database covers all
countries and contains over eleven million placenames that are available for
download free of charge.
##
data_url
## 1
https://biogeo.ucdavis.edu/data/gadm3.6/gadm36_gpkg.zip
## 2
https://www.naturalearthdata.com/http//www.naturalearthdata.com/download/10m/cultura
l/ne_10m_admin_1_states_provinces.zip
## 3
http://download.geonames.org/export/dump
##             logo_path    version source_release_date date_accessed
## 1     images/gadm.png        3.6          2020-04-15    2020-04-15
## 2 images/natearth.png      4.1.0          2021-06-02    2021-06-02
## 3 images/geonames.png 2020-04-21          2020-04-21    2020-04-21

metadata$version

##   db_version build_date code_version
## 1        2.2 2021-09-27          1.7
##
version_comments
## 1 Includes country-as-state & state-as-country, with related bugfixes and
backward-compatible database changes

metadata$acknowledgments

##       collaborator_name
## 1                  BIEN
## 2                 NCEAS
## 3 University of Arizona
## 4                   NSF
##                                  collaborator_name_full
## 1            The Botanical Information and Ecology Network
## 2 The National Center for Ecological Analysis and Synthesis
## 3                                 The University of Arizona
```

```
## 4                                The National Science Foundation
##                      collaborator_url description        logo_path
## 1 https://bien.nceas.ucsb.edu/bien/          NA  images/bien.png
## 2       https://www.nceas.ucsb.edu/          NA images/nceas.png
## 3          https://www.arizona.edu/          NA    images/UA.png
## 4              https://www.nsf.gov/          NA   images/nsf.png
```

```
metadata$citations
```

```
##          source
## 1          gnrs
## 2          GADM
## 3 Natural Earth
## 4      geonames
##
citation
## 1 @misc{gnrs, author = {Boyle, B. L. and Maitner, B. and Barbosa, G. C. and
Enquist, B. J.}, journal = {Botanical Information and Ecology Network}, title =
{Geographic Name Resolution Service}, year = 2021, url =
{https://gnrs.biendata.org/}, note = {Accessed Jun 02, 2021}}
## 2
@misc{gadm, author= {{University of California, Berkeley, Museum of Vertebrate
Zoolog}}, title = {Global Administrative Areas}, url = {https://gadm.org/}, note =
{Accessed Apr 15, 2020}}
## 3             @misc{naturalearth, author= {Kelse, Nathaniel Vaughn and Patterson,
Tom and Furno, Dick and Buckingham, Tanya and Springer, Nick and Cross, Louis},
title = {Natural Earth}, year = 2021, url = {https://www.naturalearthdata.com/},
note = {Accessed Jun 02, 2021}}
## 4
@misc{geonames, author= {{Geonames}}, title = {Geonames}, url =
{https://www.geonames.org/}, note = {Accessed Apr 21, 2020}}
```
