# Supplementary material for: Geographic name resolution service: A tool for the standardization and indexing of world political division names, with applications to species distribution modeling
Source: PLoS One. 2022 Nov 14;17(11):e0268162. doi: 10.1371/journal.pone.0268162 (PMC9662723; doi:10.1371/journal.pone.0268162)
Supplement: S6 Appendix — (PDF) [file pone.0268162.s006.pdf]

S6 Appendix: GNRS name resolution results for countries, states and counties

Data and code for replicating these results are available from doi:10.5281/zenodo.6370837.

Name match completeness and methods

The methods by which the GNRS resolved names differed among the three political division levels. At the country level, 210 (14.8%) of the 1416 unique verbatim country name strings, representing 45,574,902 (16.8%) of total observations, matched exactly to country names in the database, without the need for further resolution by the GNRS (Table S1). Of the remaining 777 (55.0%) verbatim country names requiring resolution by the GNRS, 296 were valid ISO-2 and ISO-3 codes rather than alternate names or misspellings. These 506 correct country names and codes were linked to 267,642,747 observations—the vast majority (98.8%) of the observations bearing a non-null country name (Table S2).

Table 1. Counts and percentages of total verbatim country-, state- and county-level names by different categories of resolution by the GNRS. “Already correct”: verbatim name matched exactly to a political division name in the GADM database, as submitted; “Resolved”: name matched to a political division in the GADM database, after resolution via codes, alternate names or fuzzy matching by the GNRS; “Matched”: All names matched by the GNRS (previous two categories combined); “Not matched”: no match found to a political division in GADM. Denominator for “% not matched” for political is the total unique political division names at that level; the denominator for “% not matched” for observations is all observations.

Level	Total verbatim names	Already correct	% Already correct	Resolved	% Resolved	Matched	% Matched	Not matched	% Not matched
**Political divisions:**									
country	1,416	210	14.83	777	54.87	987	69.70	429	30.30
state_province	119,901	1,225	1.02	39,966	33.33	41,191	34.35	78,710	65.65
county_parish	299,178	7,593	2.54	116,273	38.86	123,866	41.40	175,312	58.60
**Observations:**									
country	270,913,739	45,574,902	16.82	225,075,537	83.08	270,650,439	99.90	263,300	0.10
state_province	129,192,745	20,008,946	15.49	92,313,269	71.45	112,322,215	86.94	16,870,530	6.22
county_parish	111,983,421	16,138,042	14.41	23,571,465	21.05	39,709,507	35.46	72,273,914	26.65

Table 2. GNRS match methods for country names, with counts of unique names matched and numbers of linked species observations.

Match method	Verbatim countries	Percent of verbatim countries	Observations	Percent of observations

Exact alternate name	101	10.31	2,541,094	0.94
Exact alternate name, state-as-country	12	1.22	33,136	0.01
Exact ascii name	6	0.61	82,152	0.03
Exact standard name	266	27.14	45,624,773	16.86
Exact standard name, state-as-country	16	1.63	215,116	0.08
Fuzzy alternate name	128	13.06	29,108	0.01
Fuzzy alternate name, state-as-country	10	1.02	3,292	0.00
Fuzzy standard name	166	16.94	36,796	0.01
Fuzzy standard name, state-as-country	7	0.71	4,408	0.00
Inferred from country-as-state	15	1.53	4,768	0.00
ISO-3 code	46	4.69	186,554	0.07
ISO-2 code	250	25.51	221,881,291	81.98
Wildcard alternate name, state-as-country	21	2.14	7,951	0.00

The main value of the GNRS was evident at the state- and county levels, where only 1-2% of verbatim names matched directly to GADM administrative units (Table S1). At the state level, only 184 of the 41,452 verbatim names ultimately successfully matched were ISO, HASC or FIPS codes. The vast majority were names, most (31,530) of which were variant spellings or misspellings requiring some degree of required fuzzy matching to resolve (Table S3).

Table 3. GNRS match methods for state names, with counts of unique names matched and numbers of linked species observations.

Match method	Verbatim states	Percent of verbatim states	Observations	Percent of observations
Alternate code	49	0.12	305,340	0.26
Exact Alternate name	1,880	4.54	10,893,078	9.29
Exact Alternate name, country-as-state	61	0.15	862	0.00
Exact ascii name	599	1.45	489,669	0.42
Exact ascii short name	3,020	7.29	21,441,243	18.29
Exact name	4,061	9.80	68,643,775	58.56
Exact standard name, country-as-state	39	0.09	3,838	0.00
Exact standard name, county-as-state	29	0.07	23,927	0.02
Full alternate code	4	0.01	105	0.00
FuzzyAlternate name	1,676	4.05	3,358,337	2.87

Fuzzy ascii name	307	0.74	7,881	0.01
Fuzzy ascii short name	1,761	4.25	144,338	0.12
Fuzzy standard name	3,715	8.97	1,550,492	1.32
Fuzzy standard name, country-as-state	1	0.00	1	0.00
Fuzzy standard name, county-as-state	8	0.02	2,863	0.00
HASC/FIPS/ISO codes, country-as-state	6	0.01	28	0.00
ISO code	174	0.42	45,473	0.04
Wildcard Alternate name, county-as-state	18	0.04	95	0.00
Wildcard alt name	23,393	56.45	5,405,712	4.61
Wildcard alt name, country-as-state	8	0.02	39	0.00
Wildcard alt verbatim name	411	0.99	8,664	0.01
Wildcard state-in-county-field	232	0.56	4,887,526	2

At the county level, only 3 of the 123,966 verbatim names ultimately resolved were codes (ISO) as opposed to names. 57,317 names (46.2%, representing 9.7% of county-level observations) required fuzzy matching (Tables S1 & S4).

Table 4. GNRS match methods for county (admin2) names, with counts of unique names matched and numbers of linked species observations.

Match method	Verbatim counties	Percent of verbatim counties	Observations	Percent of observations
Exact alternate name	2,013	1.62	593,650	1.50
Exact alternate name, state-as-county	1	0.00	2	0.00
Exact ascii name	7,921	6.39	855,021	2.15
Exact ascii short name	17,615	14.21	21,886,901	55.12
Exact name	39,096	31.54	12,500,435	31.48
Exact standard name, state-as-county	44	0.04	215	0.00
Fuzzy alternate name	296	0.24	8,628	0.02
Fuzzy ascii name	993	0.80	54,507	0.14
Fuzzy ascii short name	3,030	2.44	153,520	0.39
Fuzzy standard name	18,895	15.24	1,335,804	3.36
Fuzzy standard name, state-as-county	9	0.01	54	0.00

ISO code	3	0.00	33	0.00
Wildcard alternate name, state-as-county	67	0.05	449	0.00
Wildcard alt name	33,457	26.99	2,306,458	5.81
Wildcard alt verbatim name	526	0.42	14,509	0.04

Unmatched names

At the country level, 429 unique country names string (30.0% of total country names, representing >0.1% of observations) remained unresolved. We identified eight main classes of issues preventing resolution of country names (Table S5). Four of these ("Not a country", "Multiple countries", "Notes in country field", and "Non geographic data or unknown") are inherently unresolvable. Another issue ("Historical country not in GADM") is in practice, unresolvable without additional information from locality descriptions, lower-level political units or dates of observation. The three remaining issues ("Country abbreviations not in reference databases", "Information in addition to country", and "Untranslated or corrupted character codes") are potentially resolvable and may be addressed in future releases of the GNRS. Examples of unresolved country names are provided in Table S6.

Table 5. Examples of the common types of errors found in country names.

**Type of issue**	**Examples**
Not a country	"America do Norte", "ANTILLAS HOLANDESAS", "Bismarck Archipelago", "Budapeste", "Europa", "Europe", "Indochina", "Malaysian Borneo", "Sicily", "Tokyo", "Washington State"
Multiple countries	"Brasil/France/Suriname", "Thailand, Peninsular Malaysia"
Notes in country field	"Desconhecido", "Flora Von Hessen-Nassau, Cassel", "[Illegible]", "invasora", "Unknown"
Country abbreviations not in reference databases	"E.U.A.", "EUA"
Information in addition to country	"Brazil (Acre, Amazonas, ParÃ¡), Colombia (Amazonas), Ecuador (Napo, Pastaza), Peru (Amazonas, HuÃ¡nuco, JunÃn, Loreto), Venezuela (Amazonas)", "Indonesia (Sumatra, Java, Krakatau)"
Untranslated or corrupted character codes.	"Colômbia", "GrÃ©cia", "LibÃ©ria", "SuÃ©cia", "PerÃº"
Historical country not in GADM	"Czechoslovakia","Yugoslavia", "YUG"
Non geographic data or unknown	"Calopogon tuberosus (L.) B.S.P.", "1938", "#?#", "4US"

Table 6. Examples of unresolved verbatim country names from biodiversity occurrence records in the BIEN database.

Country (verbatim)
Guiné
GuinÃ© Portuguesa
HavaÃ
Hawai
hawaii
Hawaii
Hawaiian Isl
Hawai & Pacific Islands
Helvetia
Howland-Baker Isl
Hungria Ocidental
IÃªmen
IdonÃ©sia
Ignorado
Ikoma
Ilha Maurigma
Ilhas Sandwich
illegible
[Illegible]
illegible1
IndÃa
India Ocidental
India Oriental
Indochina
invasora
IougoslÃ¡via
Irian Jaya
IslÃ¢ndia
ItÃ¡lia
IugoslÃ¡via
JA
Japão
JapÃ£o
JapÃ£o e China
japna
JordÃ¢nia
Juan Fernandez Isl
Kalimantan
KasaquistÃ£o

Kashmir
Kelantan
Kengo
Kerguelen
Kibu
Kihunda
Kriti
KS
Kunene-Zambeze

At the state/province level, the 78,710 names (65.6% of state-level PDCs) not resolved to state were dominated by a largely different set of issues than those found at the country level. The most commonest error was locality descriptions in the “state” field. To obtain a conservative estimate of the number of locality descriptions in the state field, we searched for long strings containing ≥6 words. We found 15,764 rows satisfying this condition (14.6% of the total PDCs, or 22.9% of unresolved PDCs). By comparison, only 89 out of 42,395 standard and alternate state/province names in the GNRS database contain ≥6 words. The next most common error was the presence of county-level political divisions in the state field. For example, “USA, Millard County” or “Peru, Província de Trujillo” (first-level political divisions in Peru are Departaments). Cases of truly unresolved states were relatively rare compared to the preceding issues. In the case of Mexico, of the 2,223 distinct unresolved state-level strings, only ~20 values appeared to be truly misspelled and, therefore potentially resolvable state names. Thus, most state-level strings not resolved by the GNRS appeared to be unresolvable non-state data. Examples of unresolved state values are shown in Table S7.

Table 7. Examples of unresolved values in the state field where country=”Mexico”, from biodiversity occurrence records in the BIEN database.

**State (verbatim)**
0.1km E Conejos, Puente Nacional Province
0.1km E of Cardenas, Cardenas Province
0.1km E Playa Brisas del Mar
0.1km NE Frontera, Frontera Province
0.1km NE Paso Dona Juana, Palmasola Province
0.1km S of Candelaria, El Carmen Province
0.1km S of Corralito, Oxchuc Province
0.1km S Villa Madero, Champoton Province
0.2km E of Cordoba
0.2km N of Playa Chalchihueca, Antigua Province
0.2km S Champoton-Escarcega, Champoton Province
0.2km S of Champoton-Escarcega, Champoton Province
0.5km E Emiliano Zapata, Villa Corso Province
0.5km N of Comalcalco-Paraiso, Comalcalco Province
100 km S of junction of Route 130 and road to Nvo. de Toluca, near La Puerta.
115 km E Hermosillo.
1.1 km N of town square of El Porvenir on road to Siltepec.

1.1 km N of town square of El Porvenir, on road to Siltepec, ca 50 m W of road, SSP 1:250,000-scale map D15-2 (very close to Spooner et al. collection 4216 collected in 1988)
11km west of Durango, along Route 40
12 km from Dolores Hidalgo on Guanajuato road, Hernandez.
1.2km northwest of Alamos.

Finally, of the 299,178 unique PDCs potentially resolvable to the county level, 123,866 (41.4%) were fully matched (Table S1). Issues observed for the remaining 175,312 (58.6%) unmatched county-level names were similar to those observed with state level names, with two exceptions: locality descriptions were less frequent (although still common), and more names appeared to be variants of genuine level 2 political divisions. Of the latter, a common class of non-matching county name included non-standard variants or abbreviations of admin2 type categories, such as "Prov.", "Distr.", "Cty." (although the GNRS removes many political division type category names prior to matching, its reference list is not complete and cannot contain all potential abbreviations). A second class of unmatched county name variant particularly common in Mexico were valid county level names preceded by a saint name. For example, the unresolved municipio "San Andres Tlalnehuayocan" in the state of Veracruz is an alternate saint name for "Tlalnelhuayocan". While the latter appears in the GADM/GeoNames reference database, the former does not. Table S8 lists a sample of unresolved county-level names for Mexico.

Table 8. Examples of unresolved Mexican municipio (admin_2, county-level) names from biodiversity occurrence records in the BIEN database, showing alternative saint name prefixes that complicate matching.

Country	State	County/parish (verbatim)
Mexico	Veracruz	San Agustin Loxicha
Mexico	Veracruz	San Agustín Metzquititlán
Mexico	Veracruz	San Andres Thamehuayocan
Mexico	Veracruz	San Andres Tlalnehuayocan
Mexico	Veracruz	San Andrés Tlalnehuayocan
Mexico	Veracruz	San Andres Tlalnehuayopan
Mexico	Veracruz	San Jose Tenango
Mexico	Veracruz	San Juan Atepec
Mexico	Veracruz	San Juan Bautista Tuxtepec
Mexico	Veracruz	SAN JUAN DEL RIO
Mexico	Veracruz	SAN JUAN GUICHICOVI
Mexico	Veracruz	San Juan Huchicobi
Mexico	Veracruz	San Juan Huichicobi
Mexico	Veracruz	San Juan Lejarcia
Mexico	Veracruz	SAN LORENZO
Mexico	Veracruz	SAN MARTIN
Mexico	Veracruz	San Martn
Mexico	Veracruz	San Miguel
Mexico	Veracruz	SAN MIGUEL
Mexico	Veracruz	San Miguel el Soldado
Mexico	Veracruz	San Miguel El Soldado

Mexico	Veracruz	San Miguel Soyaltepec
Mexico	Veracruz	SAN MIGUEL TLAPUALA
Mexico	Veracruz	San Pedro Sotepan
Mexico	Veracruz	Santa Catarina
Mexico	Veracruz	Santa Maria Chimalapa
Mexico	Veracruz	SANTA MARIA CHIMALAPA
Mexico	Veracruz	SANTA RITA
Mexico	Veracruz	Santiago Jamiltepec
Mexico	Veracruz	Santo Domingo IngenioTemascal
